# Access to Healthcare Interpreter Services: Where Are We and Where Do We Need to Go?

**DOI:** 10.3390/ijerph7072838

**Published:** 2010-07-12

**Authors:** Alexander Bischoff, Patricia Hudelson

**Affiliations:** 1 Department of Community Medicine and Primary Care, Geneva University Hospitals, Geneva, Switzerland; E-Mail: patricia.hudelson@hcuge.ch; 2 Institute of Nursing Science, University of Basel, Basel, Switzerland

**Keywords:** language barriers, migration, diversity, interpreting

## Abstract

Due to international migration, health care professionals in Switzerland increasingly encounter language barriers in communication with their patients. In order to examine health professionals’ attitudes and practices related to healthcare interpreting, we sent a self-administered questionnaire to heads of medical and nursing departments in public healthcare services in the canton of Basel-Stadt (N = 205, response rate 56%). Strategies used to communicate with foreign-language speaking patients differed, depending on the patient’s language. While nearly half of respondents relied on patients’ relatives to translate for Albanian, Tamil, Bosnian, Croatian, Serbian, Portuguese and Turkish, a third did so for Spanish, and a fourth did so for Arabic. Eleven percent relied on professional interpreters for Spanish and 31% did so for Tamil and Arabic. Variations in strategies used appear to mainly reflect the availability of bilingual staff members for the different languages. Future efforts should focus on sensitizing health professionals to the problems associated with use of *ad hoc* interpreters, as well as facilitating access to professional interpreters.

## 1. Background

Increasing linguistic and cultural diversity due to migration and international mobility has challenged the Swiss health care system. In the canton of Basel-Stadt, for example, 31% of the population is of foreign nationality and about 63% of the foreign population speaks a language other than German at home [1].

The challenges to health care posed by linguistic and cultural diversity have been extensively described [2,3], and the importance of ensuring qualified interpreters to ensure adequate patient-provider communication has been well established [4]. Nonetheless, a number of studies have indicated that health professionals continue to rely on untrained interpreters (such as bilingual colleagues and patients’ family or friends), despite the evidence that such practices are associated with poor quality of communication and care and breaches of confidentiality [5,6].

The lack of access to trained interpreters can explain to some extent the reliance on *ad hoc* interpreters. A national survey conducted in 1999 of 244 public and private internal medicine and psychiatric clinics and hospitals in Switzerland found that only 17% of services had access to professional interpreters [7]. At that time, most services relied on patients’ relatives (79%), bilingual health workers (75%) or non-medical staff (43%) to provide linguistic assistance. However, access to trained community interpreters has improved significantly since then, thanks to the creation of several community interpreter agencies, as well as the creation of a national community interpreters’ association and the development of a training program leading to a Certificate in Community Interpreting. In Basel-Stadt, interpreters are provided by “Linguadukt”, an interpreter agency run by Swiss Interchurch Aid [8].

In order to explore health professionals’ attitudes and practices regarding communication with patients who speak little or no German, and investigate how these may have changed over time, we conducted a survey in public healthcare institutions in Basel, Switzerland. This survey was part of a larger study, which also looked at interpreting practices in Geneva [9]. In this paper, we describe current practices and perceptions regarding health care interpreting, and analyze the on-going challenges to ensuring access to and use of professional interpreter services for allophone patients (*i.e.*, people who do not speak the local language [10]).

## 2. Methods

We developed a self-administered questionnaire consisting of 23 questions. The same questionnaire was used in both Geneva and Basel. The questions were worded in both German and French by the bilingual researcher team. Translation and back translation procedures were checked for accuracy by staff at the Swiss Forum for Population and Migration Studies, a research institute with vast experience in conducting bilingual surveys.

The questionnaire was mailed to all head doctors and nurses of the clinical services in each of ten hospital clinical departments in the canton of Basel-Stadt (N = 205). The departments include: internal medicine, surgery, gynaecology/maternity, psychiatry, paediatrics, ophthalmology, dermatology, ORL (oto-rhino-laryngology), geriatrics and the outpatient health services department comprising school health, occupational health, prison medicine, and substance abuse care. In a cover letter explaining the purpose of the study, these individuals were asked to either answer the questionnaire themselves or to ask a colleague of the same profession in their service to answer it. Data collection was carried out between March and November 2004.

The questionnaire asked about respondents’ socio-demographic and professional characteristics, characteristics of the clinical service in which they worked, their use of different linguistic assistance strategies in their current clinical service, and their opinions regarding the impact of interpreter services on their work and on immigrant patients’ integration into Swiss society. Integration is defined as “equal opportunity of access to societal and economic resources being granted to Swiss citizens and foreign residents (...). Key elements here are living together on the basis of common fundamental values and modes of behavior, informing foreigners about Switzerland’s institutions, laws and living conditions and the creation of conditions conducive to equal opportunities, and participation in the life of society [11]. Integration policies aim at “increasing access to and effective utilization of health care by the immigrants’ communities minimizing language, financial, administrative barriers” [12].

In our study, the term “non-Swiss patients” refers to any category of foreigner (immigrants, asylum seekers, refugees, foreign workers, *etc.*) living in Switzerland but without a Swiss passport. We use the term “professional interpreter” to refer to agency community interpreters (the primary source of professional interpreters in Switzerland), as contrasted with ad hoc interpreters. We defined three categories of *ad hoc* interpreters: bilingual employees, patients’ relatives or friends, and untrained volunteer interpreters (who are neither hospital employees nor family/friends of the patients). Respondents were asked to indicate which categories of interpreters they used for each of a list of patients’ languages. Since some respondents chose more than one option for a single language, and not all responded for all languages, the total Ns for each language vary (Table 1). Descriptive analyses (frequency distributions and cross-tabulations) were carried out using SPSS 16.0.

## 3. Results

One hundred and fourteen questionnaires were completed and returned, representing a 56% response rate. The mean estimated percent of non-Swiss patients in respondents’ clinical services was 33%, but varied widely (standard deviation 20.0). The mean estimated percent of allophone patients was 18% (standard deviation 15.2).

The majority of respondents reported using interpreters (either professional or *ad hoc*) only a few times a year (54%), while 15% said they used interpreters more than once a month, 13% said they used interpreters about once a month, and 16% reported never using an interpreter.

The strategies used most frequently to overcome language barriers varied according to the language in question (Table 1). About a third of respondents used professional interpreters most often for Tamil and Arabic. For Turkish and Albanian, 25% of respondents used professional interpreters most often, while around 20% of respondents relied primarily on professional interpreters for Bosnian, Croatian and Serbian.

For Portuguese and Spanish, only 16 and 11% respectively used professional interpreters most often. The use of bilingual employees varied from 51% (Spanish) to 7% (Arabic), the average being 18%. Between 7 and 17% of respondents used untrained volunteer interpreters most often. Patients’ relatives and friends are heavily relied upon in all languages, ranging from Arabic (24%) to 49% (Albanian).

Despite their relatively infrequent use of professional interpreters, respondents felt that interpreters had a positive effect on their ability to provide quality of health care to immigrants and on immigrants’ social integration. Ninety nine percent of respondents rated as “somewhat true” or “perfectly true” the statement that patient-provider understanding is improved with interpreter use; 97% rated as “somewhat true” or “perfectly true” that interpreters help them to more effectively communicate instructions to patients; eighty-one percent agreed that professional interpreters help them to better understand their patients’ reality, and 68% felt that interpreters helped reduce conflicts with their patients.

A large majority also felt that professional interpreters were beneficial for immigrant patients. Ninety percent rated as “somewhat true” or “perfectly true” that interpreters ensure immigrants are well informed, and help them to know their rights (72%). Nonetheless, only 22% of respondents agreed that professional interpreters help immigrants to integrate into society by increasing patients’ autonomy. Thirty-one percent rated as “somewhat true” or “perfectly true” that immigrants could become too dependent on interpreters and 36% thought that the use of interpreters prevented patients from learning the local language.

## 4. Discussion

Our study examined how public health care institutions use interpreter services in one region of Switzerland. Language barriers were common: respondents estimated that up to 18% of patients in their institutions had limited ability to communicate in German, and most had used interpreters to communicate with these patients. However, *ad hoc* interpreters were relied on heavily, especially for certain language groups (Albanian, Tamil, Bosnian). Our data suggest that respondents’ first choice is to use *ad hoc* interpreters, either family members or bilingual staff, and to call a professional interpreter only when these strategies are unavailable. This pattern was previously identified ten years ago in a national survey on interpreter use in Switzerland [7]. At that time, few professional interpreting services were available. However, *ad hoc* interpreter use continues, despite the fact that professional interpreter services have become increasingly available.

Preference for *ad hoc* interpreters has also been found in a number of other countries including Austria [13], Germany [14], Norway [15], UK [16], Ireland [17], Australia [18], USA [19], Canada [20] and South-Africa [21]. While lack of awareness of the risks associated with *ad hoc* interpreter use [22–25] may play a role, practical issues are an important influence on health care professionals’ approach to dealing with language barriers. Diamond *et al.* [6] found that physicians used *ad hoc* interpreters even though they believed that quality of care could be compromised. They tended to normalize this practice, emphasizing that practical and time constraints limited their ability to call on professional interpreters.

It is interesting to note that bilingual employees were used more often than family members for Spanish, and that use of bilingual staff for Portuguese and Serbian was also relatively high. This pattern most likely reflects the greater availability of bilingual hospital staff for these languages. We found a similar preference for bilingual staff in the Geneva arm of our study [9]. This indicates that family members may be the first strategy tried when bilingual staff are not available, but that bilingual staff are preferred. Their language skills may be superior to those of family members, and collaboration may be perceived to be easier due to their medical and/or institutional knowledge.

There are many potential practical and financial benefits to identifying and using bilingual health care staff to double as interpreters, and such programs have been implemented in a number of settings [26,27]. This strategy can be integrated into existing clinical routines, and has fewer visible costs than professional agency interpreters. However, there are invisible costs involved with removing a staff member from one role to fulfil another [28], and bilingual staff should ideally receive training in interpreting, as bilingualism is insufficient to ensure adequate interpreting skills [29].

Our study has two limitations. First, the larger study was carried out in only two Swiss cities, and therefore results may not be generalizable to other settings. Second, we had a 44% non-response rate, with no data on non-responders, and therefore cannot say to what degree our results reflect non-response bias. Nonetheless, our study provides us with a look at attitudes and practices regarding interpreter use in Basel, and suggests areas for improvement.

## 5. Conclusions

Making professional interpreter services available to hospital staff does not automatically lead to a decrease in use of *ad hoc* interpreters. There is a need to raise clinicians’ awareness of the risks and benefits of different interpreting strategies available to them, and facilitate use of professional interpreting services through information and training.

Finally, with regard to social integration of immigrant communities, we found that respondents rated the beneficial impact of interpreters in general as very high. However, there was one exception: only one in five respondents found that interpreters help immigrants to integrate into the society by “increasing patients’ autonomy”. This is in our view an area where the integration policy agenda [12] should be pushed. Therefore, decision makers, both at institutional and political level, should be sensitized to the fact that professional interpreters are an essential component in the quality care for diverse patient populations.

## Figures and Tables

**Table 1:** Interpreting strategy used most often, by language, as reported by respondents.

**Patient’s primary language**	**Professional interpreters**	**Untrained volunteers**	**Bilingual employees**	**Patients’ relatives/friends**

Tamil (N = 65)	31%	14%	12%	46%
Albanian (N = 99)	25%	14%	12%	49%
Bosnian (N = 91)	22%	10%	22%	46%
Serbian (N = 101)	19%	10%	32%	39%
Croatian (N = 101)	22%	9%	29%	41%
Turkish (N = 91)	25%	11%	27%	38%
Arabic (N = 100)	31%	17%	7%	24%
Portuguese (N = 98)	16%	15%	30%	40%
Spanish (N = 107)	11%	7%	51%	32%

## References

[1] Grillon N Thommen M Kennzahlen zur Integration von Ausländerinnen und Ausländern in Basel-Stadt 2006, Bericht im Auftrag des Wirtschafts- und Sozialdepartements Basel-Stadt Wirtschafts- und Sozialdepartements Basel-Stadt Basel, Switzerland 2008

[2] Smedley BD Stith AY Nelson AR Unequal Treatment Confronting Racial and Ethnic Disparities in Health Care The National Academies Press Washington, DC, USA 2003 25032386

[3] Derose KP Escarce JJ Lurie N Immigrants and health care: sources of vulnerability Health Aff. (Millwood) 2007 26 1258 1268 17848435 10.1377/hlthaff.26.5.1258

[4] Karliner LS Jacobs EA Chen AH Mutha S Do professional interpreters improve clinical care for patients with limited english proficiency? A systematic review of the literature Health Serv. Res 2007 42 727 754 17362215 10.1111/j.1475-6773.2006.00629.x PMC1955368

[5] Flores G Laws MB Mayo SJ Zuckerman B Abreu M Medina L Hardt EJ Errors in medical interpretation and their potential clinical consequences in pediatric encounters Pediatrics 2003 111 6 14 12509547 10.1542/peds.111.1.6

[6] Diamond LC Schenker Y Curry L Bradley EH Fernandez A Getting by: underuse of interpreters by resident physicians J. Gen. Intern. Med 2009 24 256 262 19089503 10.1007/s11606-008-0875-7 PMC2628994

[7] Bischoff A Tonnerre C Eytan A Bernstein M Loutan L Addressing language barriers to health care, a survey of medical services in Switzerland Soz. Praventivmed 1999 44 248 256 10674317 10.1007/BF01358973

[8] Hilfswerk der Evangelischen Kirchen der Schweiz (HEKS; Swiss Interchurch Aid) HEKS Linguadukt beider Basel(available online: http://www.heks.ch/de/schweiz/regionalstelle-beider-basel/linguadukt-basel/details/ (accessed on 13 June 2009).

[9] Bischoff A Hudelson P Communicating With Foreign Language - Speaking Patients: Is Access to Professional Interpreters Enough? J. Travel Med 2010 17 15 20 20074097 10.1111/j.1708-8305.2009.00314.x

[10] Bischoff A Perneger TV Bovier PA Loutan L Stalder H Improving communication between physicians and patients who speak a foreign language Br. J. Gen. Pract 2003 53 541 546 14694667 PMC1314645

[11] Saladin P Bühlmann R Dahinden D Gall Azmat R Ebner G Wohnhas J Diversity and Equality of Opportunity. Fundamentals for Effective Action in the Microcosm of the Health Care Institution FOPH—Federal Office of Public Health, in collaboration with H+ Swiss Hospital Association Bern, Switzerland 2007

[12] Bollini P Pampallona S Wanner P Kupelnick B Pregnancy outcome of migrant women and integration policy: a systematic review of the international literature Soc. Sci. Med 2009 68 452 461 19042065 10.1016/j.socscimed.2008.10.018

[13] Pöchhacker F Language barriers in Vienna Hospitals Ethn. Health 2000 5 113 119 10984829 10.1080/713667449

[14] Borde T Albrecht N-J Innovative Konzepte für Integration und Partizipation. Bedarfsanalyse zur interkulturellen Kommunikation in Institutionen und für Modelle neuer Arbeitsfelder IKO—Verlag für Interkulturelle Kommunikation Frankfurt am Main, Germany 2007

[15] Kale E Syed HR Language barriers and the use of interpreters in the public health services. A questionnaire-based survey Patient Educ Couns 2010 doi:10.1016/j.pec.2010.05.002 10.1016/j.pec.2010.05.002 20542656

[16] Gerrish K Chau R Sobowale A Birks E Bridging the language barrier: the use of interpreters in primary care nursing Health Soc. Care Commun 2004 12 407 413 10.1111/j.1365-2524.2004.00510.x 15373819

[17] MacFarlane A Dzebisova Z Karapish D Kovacevic B Ogbebor F Okonkwo E Arranging and negotiating the use of informal interpreters in general practice consultations: Experiences of refugees and asylum seekers in the west of Ireland Soc. Sci. Med 2009 69 210 214 19535192 10.1016/j.socscimed.2009.04.022

[18] Bonacruz-Kazzi G Cooper C Barriers to the use of interpreters in emergency room paediatric consultations J. Paediatr. Child Health 2003 39 259 263 12755930 10.1046/j.1440-1754.2003.00135.x

[19] Lee KC Winickoff JP Kim MK Campbell EG Betancourt JR Park ER Maina AW Weissman JS Resident Physicians’ Use of Professional and Nonprofessional Interpreters: A National Survey JAMA 2006 296 1050 1053 16954482 10.1001/jama.296.9.1050

[20] Rosenberg E Seller R Leanza Y Through interpreters’ eyes: comparing roles of professional and family interpreters Patient Educ. Couns 2008 70 87 93 18031970 10.1016/j.pec.2007.09.015

[21] Levin ME Overcoming language barriers S. Afr. Med. J 2006 96 1058 1060 17164935

[22] Lee LJ Batal HA Maselli JH Kutner JS Effect of Spanish interpretation method on patient satisfaction in an urban walk-in clinic J. Gen. Intern. Med 2002 17 641 645 12213146 10.1046/j.1525-1497.2002.10742.x PMC1495083

[23] Flores G The impact of medical interpreter services on the quality of health care: a systematic review Med. Care Res. Rev 2005 62 255 299 15894705 10.1177/1077558705275416

[24] David RA Rhee M The impact of language as a barrier to effective health care in an underserved urban Hispanic community Mt. Sinai J. Med 1998 65 393 397 9844369

[25] Jacobs E Chen AH Karliner LS Agger-Gupta N Mutha S The need for more research on language barriers in health care: a proposed research agenda Milbank Q 2006 84 111 133 16529570 10.1111/j.1468-0009.2006.00440.x PMC2690153

[26] Elderkin-Thompson V Silver RC Waitzkin H When nurses double as interpreters: a study of Spanish-speaking patients in a US primary care setting Soc. Sci.Med 2001 52 1343 1358 11286360 10.1016/s0277-9536(00)00234-3

[27] Bischoff A Steinauer R Nursing interpreters? Interpreting nurses? Pflege 2007 20 343 351 18357748 10.1024/1012-5302.20.6.343

[28] Drennan G Counting the cost of language services in psychiatry S. Afr. Med. J 1996 86 343 345 8693368

[29] Moreno MR Otero-Sabogal R Newman J Assessing dual-role staff-interpreter linguistic competency in an integrated healthcare system J. Gen. Intern. Med 2007 22 331 335 17957420 10.1007/s11606-007-0344-8 PMC2078538

